# Implementation research training for learners in low- and middle-income countries: evaluating behaviour change after participating in a massive open online course

**DOI:** 10.1186/s12961-021-00703-3

**Published:** 2021-04-06

**Authors:** Pascal Launois, Dermot Maher, Edith Certain, Bella Ross, Michael J. Penkunas

**Affiliations:** 1 Special Programme for Research and Training in Tropical Diseases (TDR), WHO, 20 Ave Appia, 1211 Geneva, Switzerland; 2 Student Academic Support Unit, Monash University, 900 Dandenong Road, Caulfield East, 3145 Australia; 3 United Nations University International Institute for Global Health (UNU-IIGH), UNU-IIGH Building, UKM Medical Centre, Jalan Yaacob Latif, 5600 Cheras, Kuala Lumpur, Malaysia

**Keywords:** Training, Massive open online course, Low- and middle-income countries, Kirkpatrick model

## Abstract

**Background:**

Implementation research (IR) can play a critical role in the delivery of disease control interventions, particularly in low- and middle-income countries (LMICs). The growing demand for IR training has led to the development of a range of training programmes and university courses, the majority of which can not be accessed by learners in LMICs. This article reports on the evaluation of the massive open online course (MOOC) developed by the Special Programme for Research and Training in Tropical Diseases hosted by WHO on the topic of IR with a focus on infectious diseases of poverty. This study followed the Kirkpatrick Model to evaluate training programmes with a specific focus on post-training changes in behaviour.

**Methods:**

MOOC participants were invited to take part in an anonymous online survey examining their knowledge of IR and how they applied it in their professional practice approximately 1–1.5 years after completing their course. The survey contained 43 open-ended, multiple choice and Likert-type questions. Descriptive statistics were calculated for the quantitative data and responses to the open-ended questions were thematically coded.

**Results:**

A total of 748 MOOC participants responded to the survey. The demographic profile of the survey respondents aligned with that of the MOOC participants, with nearly 70% of respondents originating from Africa. Responses to the quantitative and open-ended survey questions revealed that respondents’ knowledge of IR had improved to a large extent as a result of the MOOC, and that they used the knowledge and skills gained in their professional lives frequently and had consequently changed their professional behaviour. Respondents most often cited the problem-solving aspect of IR as a substantial area of behavioral change influenced by participating in the MOOC.

**Conclusions:**

These findings indicate that the MOOC was successful in targeting learners from LMICs, in strengthening their IR knowledge and contributing to their ability to apply it in their professional practice. The utility of MOOCs for providing IR training to learners in LMICs, where implementation challenges are encountered often, makes this platform an ideal standalone learning tool or one that could be combined with other training formats.

## Contributions to the literature

- Implementation research plays a crucial role in disease control interventions.
- Efficacious interventions are particularly needed in low- and middle-income countries in relation to infectious diseases of poverty.
- Knowledge and training in implementation research is limited in low- and middle-income countries.
- Online training, such as a MOOC, provides a proven way to educate a broad section of learners in low- and middle-income countries about implementation research.

## Background

Health systems have an ever-growing suite of evidence-based interventions at their disposal—vaccines, pharmaceuticals, diagnostic technologies—yet these interventions often do not translate into a real-world impact when introduced at scale [1]. The need to address bottlenecks in the implementation of efficacious health interventions is often greatest in low- and middle-income countries (LMICs), where health systems are underdeveloped and do not have established mechanisms to effectively collect and use locally generated information [2]. Building research capacities to identify and enact strategies for overcoming implementation challenges is increasingly important as countries strive for universal health coverage while combating the burden of infectious and noncommunicable diseases at varying levels of intensity. Researchers in LMICs are in the best position to produce locally generated evidence for priority-setting to ensure interventions remain relevant and sustainable, and are implemented optimally when scaled.

While implementation research (IR) can play a critical role in the delivery of disease control interventions, it is an area that is only recently gaining attention and support [3]. The increasing interest in IR and growing demand for IR training has led to the development of a range of training programmes and university courses [4–6], which were not widely taught until recently [5]. Yet, the majority of these formal courses remain out of reach for researchers in LMICs. In order to disseminate IR concepts widely and train public health researchers, practitioners and policy-makers in LMICs, the Special Programme for Research and Training in Tropical Diseases (TDR) hosted by WHO developed a massive open online course (MOOC), which was piloted with success in 2017 and rolled out to wider audiences in 2018 [7].

## The TDR implementation research MOOC

Massive open online courses are gaining popularity as professional development and credentialing tools [8] and can increase professional knowledge of a new topic [9] and the development of new skills [10]. MOOCs historically display low retention rates in high-income countries [11–13] although completion rates of professional development MOOCs have been found to be higher in LMICs [14–16]. Beyond learner retention in MOOCs, whether or not participants apply their learnings to their professional lives remains a substantial limitation [17, 18].

The IR MOOC examined here was developed by TDR through a series of consultations and engagements with recognized experts in IR. The focus for the MOOC is on applying IR concepts to increase the effectiveness of interventions for combating infectious diseases of poverty. The IR MOOC was developed to address the relative lack of education and training opportunities in IR in LMICs. The five-module MOOC is delivered over 6 weeks and illustrates IR concepts through a series of videos, with worked case studies that are introduced, presented and interpreted by experienced public health experts. Videos in the MOOC range from approximately 4 to 25 min in length and are divided into the five following modules: (1) What is Implementation Research? (Defining IR; assessing the appropriateness of existing disease control interventions); (2) Needs Assessment for Implementation Research (Identifying the challenges within various health settings; describing implementation bottlenecks through consultations with communities and stakeholders); (3) Designing Implementation Research (Specifying implementation research questions; designing rigorous research projects; developing new implementation strategies by working with communities and stakeholders); (4) Implementation Research Outcomes (Identifying IR outcomes and evaluating effectiveness of interventions in real-life settings); (5) Implementation Research in Practice (Planning for the scale-up of implementation and demonstrating the potential of IR in disease control programmes). Discussion forums further exploring the concepts presented in the video presentations are facilitated by TDR trainers and encourage interaction among students. The MOOC targets researchers and practitioners and has the aim to build a foundational knowledge base of IR methods, purpose and approaches [19, 20].

The IR MOOC is currently offered in English, French and Spanish, with Russian and Chinese, and Arabic versions to be offered in 2020 and early 2021 respectively. Further refinements of the MOOC are currently under development, including the development of additional, specialized modules on in-demand topics such as qualitative methods, community engagement and integrating gender and intersectionality into IR studies. In addition, although the MOOC was developed with a focus on infectious diseases of poverty, it has been adapted for strengthening IR capacity around noncommunicable diseases. [21].

## Evaluation using the Kirkpatrick model

Evaluation is critical for assessing the effectiveness and value of MOOCs [22]. The Kirkpatrick Model is widely used to evaluate training programmes and has been used previously to evaluate MOOCs for learners in high-income settings [23–27]. The model comprises four levels and examines: (1) reaction; (2) learning; (3) behaviour; and (4) results. The Kirkpatrick model provides a framework for both short- and long-term evaluations [28], with short-term evaluations typically focussing on learners’ more immediate reactions to the training and their learning. Long-term evaluations explore whether and how learners have used the training to modify behaviour and any concrete results several months to years after the training takes place.

Evaluations of the immediate reaction and learning of the IR MOOC participants showed significant improvement toward strong and very strong IR knowledge. Prior research has reported that approximately three quarters (72.3%) of surveyed participants indicated that the MOOC met their expectations and 80.9% indicated substantial improvement in IR knowledge [29].

The current paper presents an evaluation of *behaviour*. In the present context, behaviour refers to how the new knowledge, skills and attitudes are applied in practice. For this study, we examine if and how participants’ applied the concepts presented in the IR MOOC in their professional roles and responsibilities.

## Methods

This analysis of participants’ behaviour is based on online, anonymous survey data collected between November 2019 and February 2020. The survey data include both quantitative and qualitative data, whereby the qualitative data assisted in contextualizing the quantitative findings and ensured the comprehensiveness of the results. The main area under examination here is changes in professional behaviour. The quantitative and qualitative data are integrated in the presentation of findings according to these two areas; the overall areas of change are presented first using quantitative data, followed by the qualitative data that explore in further detail the themes and nuances of responses.

The survey consisted of 43 questions, which included open-end, multiple choice and Likert-type questions. Questions centred on four areas: (1) participant demographics; (2) satisfaction with the MOOC; (3) change in professional behaviour following the MOOC; and (4) results following the MOOC that affected either the participant’s professional work or their organization.

Ten participants of a previous IR MOOC were invited to pilot the survey on 23 November 2019. Of these, four responded to the invitation within the requested 3-day period. The survey was modified slightly following analysis of the responses in this pilot survey. The link to the revised survey was emailed to all IR MOOC participants from the May and October cohorts in November 2019, and three reminder emails were sent during the next 10 weeks. In total, 748 responses were recorded between November 2019 and February 2020, with a response rate of 19.4%.

Qualtrics survey software [30] was used to collect the survey data. Descriptive statistics were used to analyse and present the results of the quantitative data using Microsoft Excel (Microsoft Corp., Redmond, WA, USA). Data from the open-ended questions were thematically coded in Microsoft Excel using Braun and Clarke’s six phases [31]: (1) familiarizing oneself with the data; (2) generating initial codes; (3) searching for themes; (4) reviewing themes; (5) defining and naming themes; and (6) producing the report. Thematic analysis was based on a selection of existing literature and discussions between one of the authors (BR) and the primary coder; the same author double coded approximately 10% of the data to check for interrater reliability. Levels of agreement were high (> 90%), and any inconsistencies were resolved through discussion between the two coders. The CRe-DEPTH guidelines [32] were used to inform the reporting of this evaluation.

## Results

### Demographics of MOOC registrants and survey respondents

Table 1 summarizes and compares the demographics of the survey respondents and MOOC registrants, revealing that the survey respondents are largely representative of the two cohorts of MOOC registrants.

**Table 1:** Comparison of survey respondents’ and MOOC participants’ demographic profiles

Demographic parameters	Survey respondents	MOOC participants
*N*	748	3858
Gender	Female: 44.1% Male: 55.9%	Female: 44% Male: 57%
Age	Between 26 and 40 years: 67.5%	Between 20 and 40 years: 77.5%
WHO region	African Region: 69.4% South-East Asia Region: 12.6% Americas Region: 9.6% Eastern Mediterranean Region: 1.6% European Region: 4.1% Western Pacific Region: 2.7%	African Region: 62.4% South-East Asia Region: 17.7% Americas Region: 9.9% Eastern Mediterranean Region: 5.5% European Region: 2.8% Western Pacific Region: 2.2%
Profession	Public health researchers: 31.2% Public health officers 17.4% Students: 15.3% Teachers: 11.4% General practitioners: 9.3%	Public health researchers: 45% Public health officers: 15.5% General practitioners: 11.1% Students: 11%
Education level attained	Master’s degree: 57.1% Bachelor’s degree: 25.6% PhD/Doctorate: 17.3%	Master’s degree: 41.5% Bachelor’s degree: 24.7% PhD: 10.5% Medical Doctorate: 17.1%
Certificate of completion obtained	70.6%	Of the total initially enrolled: 30.15% Of those who completed the course: 89.2%

#### Demographics of survey respondents

The demographic profiles (Table 1) were combined for the May and October cohorts: the May cohort accounts for 45.4% of all responses and the October cohort for 54.6%. The survey respondents originated from 89 different countries, with the majority of respondents (69.4%) originated from Africa. South-East Asia was the second most represented region (12.6%). The sex of participants was nearly balanced (55.9% male) and over two thirds of participants (67.5%) were between 26 and 40 years old. Public health researchers were the most represented professional group (31.2%) followed by public health officers (17.3%), students (15.3%), teachers (11.4%) and general practitioners (9.3%). The majority of respondents held a master’s degree (57.1%), and 25.6 and 17.3% had attained a bachelor’s degree or doctorate, respectively. Of the 748 MOOC participants who took part in the survey, 525 (70.6%) had completed the required assessments to earn a certificate of completion for the MOOC and 11% indicated that they had participated in additional courses on IR following the MOOC. These additional courses included 1-day workshops and symposiums, week-long training courses, online courses, 1-year certificates and Master’s degree programmes.

#### Demographics of MOOC registrants

**Fig. 1:**
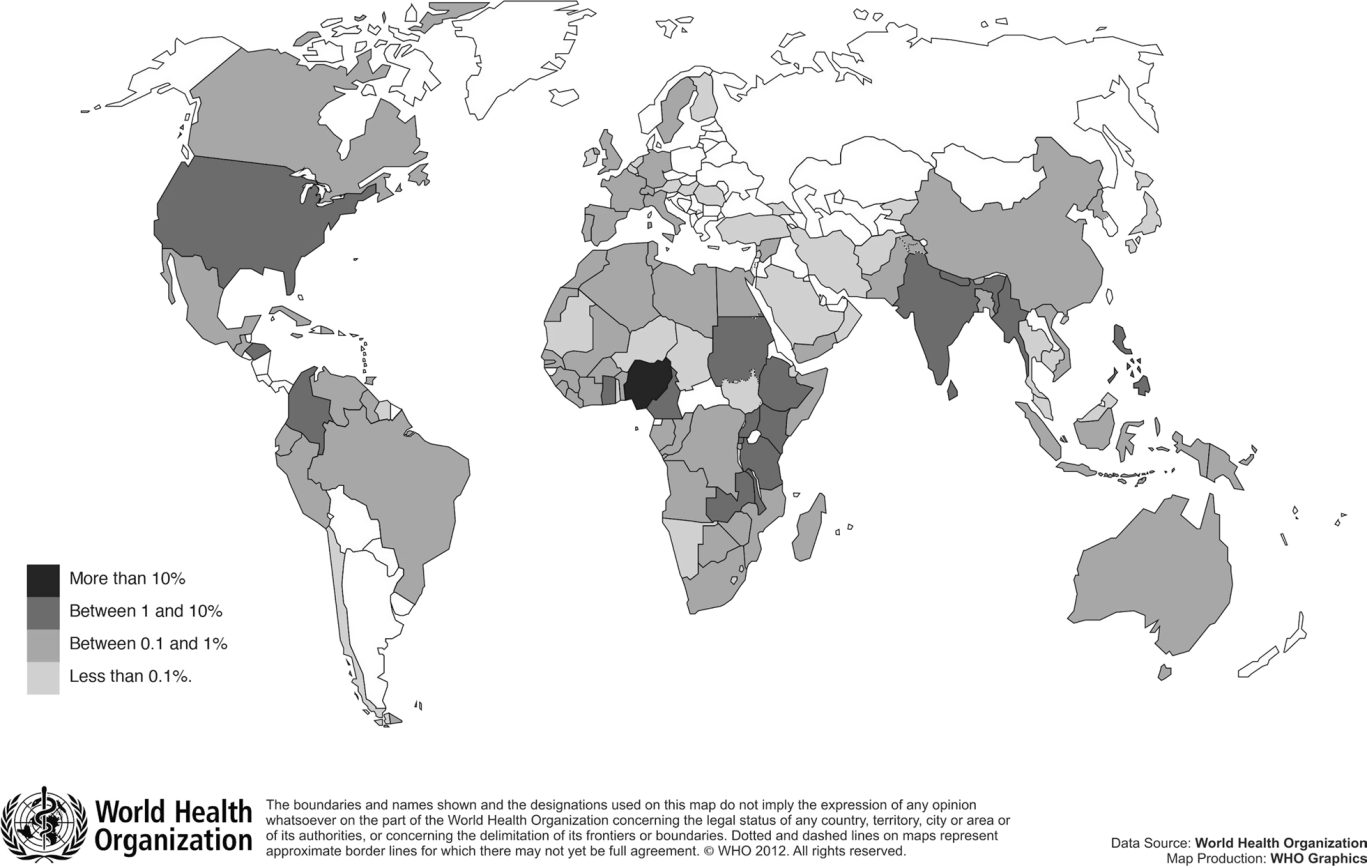
Countries of represented MOOC registrants

Of the 3858 registrants across the May and October MOOC offerings, 1163 participants (30.2%) completed the assessments required to earn a certificate of completion. The MOOC registrants came from 115 different countries, with the majority originating from the WHO African Region. The majority of registrants came from English-speaking countries (87.2%), followed by Spanish- (7.7%), French- (4.6%) and Portuguese-speaking countries (1.4%). Figure 1 shows the regions of origin for the registrants.

Compared to male participants, female participants were more often public health officers (51.6 vs. 46.5%) and students (56.8 vs. 43.3%). Men were more often public health researchers (59 vs. 41%) and general practitioners (57 vs. 43%) compared to women. See Table 1 for a profile comparison of survey participants and individuals who took part in the MOOC.

### Knowledge of implementation research leading to behaviour changes

Respondents were asked to indicate their reasons for taking the IR MOOC through a multiple choice question with an ‘Other’ option. The most commonly cited reasons included: (1) to gain knowledge and understanding of IR (20.8%); (2) to apply the knowledge and tools in research (15.1%); (3) to apply the knowledge and tools in practice (13%); (4) for self-learning purposes (11.3%); (5) to further specialize in their field (8.5%); and (6) to obtain a certificate of completion (7.4%).

The majority of respondents considered their current knowledge about IR theory (frameworks, models and concepts) to be very good (15.6%), good (58.4%) or fair (23.1%). The IR MOOC contributed to their knowledge to a very large extent (18.1%), a large extent (50.5%) or a moderate extent (26%).

In order to understand in further detail which areas respondents felt the IR MOOC had contributed to, including behavioural changes associated with their profession, they were asked what they considered as the most important insights or experiences gained from the course through an open-ended question. Of the 558 recorded responses to this question, five main themes that directly pertain to changes in behaviour were identified using Braun and Clarke’s six-phase analysis [28], namely:

1. Problem-solving aspect of IR (*N* = 71)
2. Applicability or relevance of IR (*N* = 68)
3. Research process (*N* = 58)
4. Appreciation of stakeholder and community involvement (*N* = 41)
5. Programme evaluation (*N* = 36)

The following sections discuss each of these themes in further detail and provide representative statements.

#### Problem-solving aspect of implementation research

Respondents identified the importance of IR in identifying methods for solving community health implementation issues in particular contexts. Such problem-solving skills allow participants to modify their behaviour and identify appropriate solutions in regard to IR projects. The two representative statements below illustrate this point.

“The ability to look at challenges from a broader perspective, visualising the different aspects of the problem and once identified, solutions are easily found.” (Public health officer, Cameroon, obtained certificate).

“The most important insights or experiences that I gained from this course were implementation outcomes and implementation strategy. Now I can identify [an] implementation problem and propose a way to resolve it.” (Public health researcher, Burkina Faso, obtained certificate).

Methods for identifying “bottlenecks” were conveyed throughout the MOOC, and respondents appear to have incorporated these learnings into their professional work.

#### Applicability or relevance of implementation research

Respondents’ comments indicated that they felt the MOOC IR highlighted how IR was applicable or relevant to their profession or research area. This impacted on participants’ behaviour by enabling them to apply their knowledge of the relevance of IR to specific professional situations. One participant stated:

“Stakeholders’ involvement at all the stages of implementation research/activities. It was the most important insight because it was very impactful in my professional career… I have been able to implement project [sic] much more effectively and efficiently.” (Public health researcher, Uganda, obtained certificate).

A second respondent outlined how the knowledge gained through the MOOC will positively impact on their work:

“Having participated in community-based intervention programmes in my home country Nigeria, in the past, the knowledge I acquired in the course of the training revealed the causes of most of the challenges encountered and further gave me insight on how to carry out future intervention programmes in such a way that the community benefit more.” (Student, China, obtained certificate).

The applicability of the course material was also relevant to students and teachers’ professional behaviour and outcomes as evident from the following statement:

“The course helped me to restructure my PhD research work and my teaching materials for my students.” (Student/teacher, Nigeria, obtained certificate).

Respondents could therefore use the knowledge gained from the MOOC to make practical changes in their jobs.

#### Research process

In terms of research, respondents found the MOOC helpful in explaining in detail how IR is conducted and they were able to apply this knowledge in their research behaviour. This includes designing, planning and writing IR research proposals, applying for funding, writing publications and developing research frameworks and methodologies. Representative statements include:

“How to write a protocol; how to choose between quantitative/qualitative methods and why/how to use mixed-methods.” (Public health researcher, Cameroon, obtained certificate).

“The most important insight gained is how to implement a plan accurately, communicate clear goals and anticipations, and evaluate programs fairly to learn what components are necessary to produce intended effect.” (Human resource administrator/Researcher, Nigeria, obtained certificate).

These responses reveal that respondents felt capable of developing research in IR following the MOOC.

#### Appreciation of stakeholder and community involvement

Respondents stated that the course had contributed to their understanding of the significance of community or stakeholder involvement, engagement or participation in the IR process. This understanding shaped participants’ behaviour in implementing projects. Statements include:

“I learnt that involving community is vital for the successful implementation of interventions… I have been able to critically think through the various interventions taking care not to exclude the community and community leadership.” (Public health researcher, Uganda, did not obtain certificate).

“Involve all the stakeholders from the beginning, make them partners, defining problems and providing solutions with them, improves the effectiveness of research and make it transparent, open and fun!” (Research promotion and development administrator, Colombia, obtained certificate).

As illustrated by the following statement, some respondents displayed a nuanced understanding of how strong community engagement helps maximize the chances that an intervention will succeed.

“I understood that community mobilization will assist the community to identify the disease prevalent in the locality and available interventions targeted on diseases and the place they can assess care. This will lead to community involvement and participation, meaning that they will be involved in identifying their felt need. They will also select the trusted village health workers who will be trained to be involved in providing health services to the community.” (Public health officer, Nigeria, obtained certificate).

This participant then clarifies how this has led to a change in professional behaviour with the following comments:

“I ensure that the interventions targeted at the diseases of poverty are utilized by the communities.”

“It helped me to reach out to the community and more people are assessing care in the hospital.”

These findings indicate that the MOOC was successful in educating participants about the crucial role of stakeholder and community engagement in IR and in modifying their behaviour accordingly.

#### Programme evaluation

A final theme relates to the significance of evaluating the effectiveness of implementation of intervention programs. Important learning and professional and research behaviours cited by respondents are highlighted by the three respondents’ statements below.

“How to use research tools to evaluate and improve on health programs.” (Public health officer, Cameroon, obtained certificate).

“The evaluation of existing frameworks in order to introduce interventions that will improve on the status quo.” (Student, Ghana, obtained certificate).

“How to assess the appropriateness of existing implementation strategies.” (Student, Nigeria, obtained certificate).

These responses indicate that those surveyed had engaged with the importance of evaluating an intervention’s effectiveness.

### Changes in professional behaviour

In relation to changes in respondents’ work, the majority stated that the knowledge gained from the course had been valuable to a large extent (43.2%), followed by valuable to a moderate extent (27.8%) and to a very large extent (17.9%). The majority of respondents have had use of the knowledge gained from the MOOC to a very large extent (12.6%), large extent (36.3%) or moderate extent (32%). The majority of respondents have had use of the knowledge gained from the MOOC monthly (30.6%), rarely (29.9%), daily (25.2%) or weekly (10.6%).

Respondents were asked whether they performed their role and responsibilities differently as a result of participating in the MOOC: 69.1% agreed, while 30.9% disagreed. Of the 574 respondents, 330 elaborated on their response. The most common themes cited were:

1. Research processes (*N* = 94)
2. Understanding and thinking about IR and issues related to research (*N* = 73)
3. Stakeholder and community involvement (*N* = 46)
4. Problem-solving (*N* = 31)
5. Leadership (*N* = 20)
6. Involvement in IR projects (*N* = 18)
7. Teaching and training others (*N* = 16)
8. Programme development, monitoring and evaluation (*N* = 13)

#### Research processes

The most commonly cited difference in how respondents performed their role and responsibilities differently as a result of the course related to knowledge of and ability in research processes. This includes designing, planning and identifying research problems, developing research objectives, collecting data, presenting research outcomes, writing research proposals and publications and applying for research grants. Three illustrative statements of this are presented below.

“[The MOOC] helped me in developing a research proposal for my organization, where I used knowledge from this course.” (Public health researcher, Nepal, obtained certificate).

“I produced more elaborate reports for projects, and better correspondence as I am able to identify relevant stakeholders.” (Public health researcher, Ghana, obtained certificate).

“I have by myself completely written an IR proposal which has been funded. The course gave me the idea to include in my proposal participatory action-research and mixed-methods. I do think this played in my favor and made the proposal accepted by [name of organisation].” (Public health researcher, Cameroon, obtained certificate).

Respondents’ self-reported changes in their research processes and outcomes reveal that the MOOC was successful in teaching IR research approaches.

#### Understanding and thinking about implementation research

The second most commonly found theme included respondents’ increased understanding of and thinking about IR and both broad and narrow issues related to research, which had contributed to them performing their role and carrying out responsibilities differently. Representative statements include:

“These courses have increased my knowledge in the implementation of research and allowed me to have a broad view of things.” (Student, Congo, did not obtain certificate).

“From the knowledge gained from MOOC, I have changed how I perceived research implementation.” (Internal medicine resident, Rwanda, obtained certificate).

Such statements reveal the positive impact of the MOOC in shifting and developing participants’ thinking and understanding about IR, and respondents felt that this had resulted in them performing their professional duties differently.

#### Stakeholder and community involvement

Respondents commonly cited stakeholder and community involvement as an area in which they performed their role and carried out their responsibilities differently after participating in the IR MOOC. The following statements illustrate this point.

“For all the interventions I plan, I promote the model of district-led-programming (DLP) to allow communities [to] lead the way.” (Public health officer, Uganda, obtained certificate).

“I always think of diverse stakeholders necessary for the success of a community-based intervention.” (Student, Tanzania, obtained certificate).

Comments reveal that respondents were aware of the importance of stakeholder and community context, and that including them increases the chances of the success of an intervention:

“By following participants of my research to their local areas to learn challenges they face.” (Public health researcher, Tanzania, did not obtain certificate).

“I often try to involve stakeholders in public health activities to promote acceptance.” (Public health officer, Uganda, obtained certificate).

The importance of stakeholder and community engagement was cited by respondents in relation to their understanding of IR as well as changes in their professional practice.

#### Problem-solving

Respondents reported improvements to their ability to solve IR problems as a result of the course, as illustrated by the statements below.

“The knowledge gained enables me to find simple yet innovative ways of solving problems.” (General practitioner, Ghana, obtained certificate).

“I have implemented IR in my routine activities with problem solving in mind.” (Public health officer, Nigeria, obtained certificate).

Some respondents described how the knowledge gained from the MOOC had improved their abilities as a researcher and led to positive professional outcomes.

“This is because the knowledge I acquired has transformed me into a more ardent researcher with ideas on how to identify and solve implementation problems. These ideas have contributed to designing an implementation research [study] identified by WHO/TDR experts, which gave me a chance of selection as one of the five finalists that attended [name of workshop].” (Public health researcher, Nigeria, Obtained certificate).

As problem-solving is a core component of IR, these statements by respondents reveal that the MOOC successfully communicated this point.

#### Leadership

A positive change cited by respondents concerned their increased abilities and roles in leadership. This change includes leading research teams, managing research projects, taking on additional responsibilities and contributing to policy-making. Some respondents described the roles they were able to take on as a result of the MOOC.

“Provide guidance and knowledge transfer to stakeholders and implementing partners on areas that need to be improved or strengthened in implementation of appropriate interventions, and documentation of the process.” (Public health officer, Somalia, obtained certificate).

One respondent provided an example of how this new ability contributed to their ability to lead research teams nationwide.

“As a senior research advisor I am using the skills I have acquired to develop concept notes and protocols for IR and guide research teams across the country.” (Public health researcher, Ethiopia, obtained certificate).

For others, the acquired leadership abilities assisted them to improve the implementation of public health programming.

“As a programme manager, it has helped me guide programme implementation in identifying gaps and steps/required interventions to achieve desired implementation outcomes.” (Public health officer, Nigeria, obtained certificate).

#### Involvement in implementation research projects

Respondents identified increased involvement in a range of IR projects as a result of the MOOC. This is illustrated by the following statements.

“I have conducted TB implementation research in my country.” (Research and development/private sector, Sudan, obtained certificate).

“I have been part of various teams in Nigeria developing and working on implementation research.” (Public health officer, Nigeria, did not obtain certificate)

Some respondents described how the MOOC had refreshed their knowledge and enabled them to apply it to IR projects.

“My MPH training included some coursework on implementation science (IS) but the MOOC really helped me refresh my knowledge and apply IS lens to my role at [organization name]. My role is focused on managing global health security project implementation across several countries in Africa and Asia.” (Public health officer, United States of America, obtained certificate)

#### Teaching and training others

Respondents indicated that the course had assisted them in their roles and responsibilities regarding teaching and training.

“As a programme manager, it has helped me guide programme implementation in identifying gaps and steps/required interventions to achieve desired implementation outcomes.” (Public health officer, Nigeria, obtained certificate).

Some participants stated that the course had improved their thinking around teaching and training.

“I now design and teach research in a manner that its findings could be implemented. (Teacher, Nigeria, obtained certificate).

“Greater clarity when advising students how to execute their investigations in the field of tuberculosis in the Master of Epidemiology at [university name].” (translated from Spanish) (Teacher, Colombia, obtained certificate).

Others indicated that the MOOC had increased their confidence in this area.

“I feel more confident to train others in IR.” (Public health researcher, Colombia, obtained certificate).

#### Programme development, monitoring and evaluation

The MOOC increased respondents’ skills within general programme development, monitoring and evaluation.

“I ensure that the interventions targeted at the diseases of poverty are utilized by the communities.” (Public health officer, Nigeria, obtained certificate).

“I have been able to assess bottlenecks from a systems point of view and realize how these bottlenecks can or could be solved with implementation research which helps us to understand why they are occurring in the first place.” (Student, Malawi, obtained certificate).

Some respondents indicated they were able to transfer this knowledge to other areas.

“After taking the course, I used knowledge gained in assessing the Adolescent Sexual and Reproductive Health program running in my district and I used findings to change some methods in adolescents education and care in school and at health facilities in my district.” (Public health officer, Ghana, obtained certificate).

A second participant stated:

“In that I turn every public health program (food safety programs for instance) around to detect any possible gap in its implementation and find solutions to them before getting it out to its consumers or beneficiaries.” (Regulatory officer, Ghana, obtained certificate).

Such findings are important in that they confirm the usefulness of the IR MOOC in developing participants’ ability to develop, monitor and evaluate programmes that are not focused on infectious diseases of poverty.

## Discussion

The IR MOOC sessions offered in 2018 reached the targeted audience, namely those working within public health and research in LMICs. Those surveyed were largely representative of the participants of the two sessions of the 2018 IR MOOC. Most registrants and survey respondents were located in Africa and South-East Asia where implementation challenges are often the greatest. Participants from certain countries in Africa, such as Nigeria, Cameroon and Kenya, are more represented than others due to these countries having some of the largest English-speaking populations on the continent. As the two iterations of the MOOC assessed here were presented in English and open to participants free of charge, it is expected that a large percentage of participants originated from these countries. The representation of countries may also illustrate a relationship with the number of health researchers in those countries, with countries having large researcher bases being overrepresented in the MOOC. Additionally, some of the highly represented countries, such as Ethiopia and Nigeria, possess larger numbers of institutions conducting research through external funding than many of those countries that are less represented. In Ghana, for example, TDR has strong relationships with several research and educational institutions, and the MOOC was managed by The School of Public Health at the University of Ghana, which is an institution supported by TDR. Through this support, The School of Public Health at the University of Ghana is helping institutionalize and disseminate a series of short trainings in IR throughout Africa.

The IR MOOC participants and survey respondents mainly worked as public health researchers or public health officers, and the range of individuals was broad in terms of country, profession, level of education, gender and age. These characteristics suggest that the IR MOOC was able to reach people from a variety of contexts and backgrounds who were in need of training. This is particularly relevant to IR in LMICs where health systems are often underdeveloped and there is a lack of established approaches to collecting and using locally generated information [2]. In these contexts, researchers are needed to create locally relevant evidence-based solutions to implementation challenges to ensure that interventions are relevant, sustainable and optimally delivered in order to reach the intended audience at scale.

The IR MOOC was successful in its aim of strengthening participants’ knowledge of IR. The majority of respondents considered their current knowledge of IR to be very good (15.6%) or good (58.4%) and that the IR MOOC contributed to their knowledge to a very large extent (18.1%) or a large extent (50.5%). In relation to specific areas of knowledge gained from the course, analysis of the responses to themes revealed that IR problem-solving, including the identification of bottlenecks, and the applicability or relevance of IR were the most commonly cited themes. Respondents stated that they had learnt about the importance of including stakeholders and communities throughout the IR process in order to achieve the best possible outcomes. These findings indicate that the MOOC was an effective means of educating people in IR concepts; participants reported that the MOOC contributed to their learning and they were able to retain the content for future professional use. Such insights are crucial to building research capacity to identify, develop and implement interventions that are able to overcome challenges that may hinder successful outcomes. This is particularly the case in LMICs and in relation to combating infectious diseases of poverty and the growing burden of non-communicable diseases [33].

The IR MOOC proved successful in strengthening participants’ professional practice in relation to IR as participants were able to apply the MOOC content in their daily professional lives. In terms of changes in respondents’ daily professional behaviour as a result of the IR MOOC, respondents reported using the new knowledge and skills that they gained from the course fairly frequently and that these are of professional value. These findings are similar to those of an evaluation of a MOOC on implementation science using the Kirkpatrick Model [4] which likewise revealed positive short- and long-term learning outcomes; participants indicated that they found the different theories, models and frameworks applicable to their own research and appreciated the research networks that they gained access to. The most commonly cited themes regarding changes to respondents’ roles and responsibilities include how they approach the research process and their understanding and thinking about IR and IR findings. Respondents reported improvements in how they approach problem-solving, including identifying bottlenecks, and how they approach stakeholder and community engagement with IR activities. For many, the MOOC had increased opportunities for or in leadership roles, as well as involvement in IR projects. A further positive finding was that respondents were able to transfer their learning from the IR MOOC to develop, monitor and evaluate programmes dealing with health issues that do not specifically target infectious diseases of poverty.

While the overwhelming majority of respondents’ comments were positive, not all were. Several respondents stated that they were unable to complete the MOOC due to heavy workloads or poor internet connections. Not all respondents were able to apply the knowledge and skills to a large extent, and many stated that they had faced barriers to implementing the knowledge and skills that they learnt in the course in the workplace. This is explored further in a separate article focussing on the participants’ self-reported use of the knowledge gained in the MOOC in their professional work and the barriers encountered in the workplace [34].

These findings provide positive evidence that MOOCs can be used as a tool to further knowledge of and engagement in IR. This is particularly important given that many of the available evidence-based interventions do not have significant impacts when introduced at scale [1]. Bottlenecks and a lack of local stakeholder and community engagement are a particular issue to the implementation of efficacious health interventions in LMICs, where health systems may lack established mechanisms to employ locally generated information [2]. Respondents of the IR MOOC felt that these were areas in which they had gained important knowledge and that they were able to apply this in their professional practice.

### Study limitations

A number of limitations to this study are worth noting. First, response bias may contribute to the positive results reported here, as there may be differences between those who responded to the survey request versus those who do not [35]. Survey and interview respondents are highly self-selective and this may be linked to learners with autonomous motivations for behaviour [36, 37]. The data is self-reported by participants and may therefore present validity issues. The lack of a control group is a further limitation to the study.

## Conclusion

The IR MOOC was successful in targeting people from a variety of LMIC contexts and backgrounds and in strengthening their knowledge of IR. Furthermore, learners following the MOOC were able to apply this knowledge of IR in their professional practice. As such, MOOCs can be seen as a proven method of delivering IR education to LMIC learners who may not have access to formal learning pathways, such as university courses. This is particularly relevant to learners in LMICs, where challenges in relation to implementation are often encountered. Furthermore, MOOCs can also be utilized to satisfy the increasing interest in and growing demand for IR training within the general population.

## Data Availability

The datasets used during the current study are available from the corresponding author on reasonable request.

## Abbreviations

IR: Implementation research
LMICs: Low- and middle-income countries
MOOC: Massive open online course
TDR: Special Programme for Research and Training in Tropical Diseases
